# Radio-Fluorogenic Gel Dosimetry with Coumarin

**DOI:** 10.3390/bioengineering5030053

**Published:** 2018-07-10

**Authors:** Peter A. Sandwall, Brandt P. Bastow, Henry B. Spitz, Howard R. Elson, Michael Lamba, William B. Connick, Henry Fenichel

**Affiliations:** 1 Department of Radiation Oncology, OhioHealth, 330 Glessner Ave., Mansfield, OH 44903, USA; 2 Department of Chemistry, College of Arts and Sciences, University of Cincinnati, Cincinnati, OH 45221, USA; brandtbastow@gmail.com (B.P.B.); connicwb@ucmail.uc.edu (W.B.C.); 3 Department of Nuclear Engineering, College of Engineering, University of Cincinnati, Cincinnati, OH 45221, USA; spitzh@ucmail.uc.edu; 4 Department of Radiation Oncology, College of Medicine, University of Cincinnati, Cincinnati, OH 45221, USA; elsonhr@ucmail.uc.edu (H.R.E.); lambama@ucmail.uc.edu (M.L.); 5 Department of Physics, College of Arts and Sciences, University of Cincinnati, Cincinnati, OH 45221, USA; fenichel@ucmail.uc.edu

**Keywords:** gel dosimetry, radiation dosimetry, radio-fluorogenic gel, luminescent dosimetry

## Abstract

Gel dosimeters are attractive detectors for radiation therapy, with properties similar to biological tissue and the potential to visualize volumetric dose distributions. Radio-fluorogenesis is the yield of fluorescent chemical products in response to energy deposition from ionizing radiation. This report shares the development of a novel radio-fluorogenic gel (RFG) dosimeter, gelatin infused with coumarin-3-carboxlyic acid (C3CA), for the quantification of imparted energy. Aqueous solutions exposed to ionizing radiation result in the production of hydroxyl free radicals through water radiolysis. Interactions between hydroxyl free radicals and coumarin-3-carboxylic acid produce a fluorescent product. 7-hydroxy-coumarin-3-carboxylic acid has a blue (445 nm) emission following ultra-violet (UV) to near UV (365–405 nm) excitation. Effects of C3CA concentration and pH buffers were investigated. The response of the RFG was explored with respect to strength, type, and exposure rate of high-energy radiation. Results show a linear dose response relationship independent of energy and type, with a dose-rate dependency. This report demonstrates increased photo-yield with high pH and the utility of gelatin-RFG for phantom studies of radiation dosimetry.

## 1. Introduction

Advancements in radiation therapy technology have supported study of tissue-equivalent gels containing active chemical sensors for the measurement of absorbed dose of radiation. Gel dosimeters have radiological properties similar to biological tissue, and are suitable substitutes with the potential to resolve three-dimensional dose distributions. The development of gel dosimeters was dormant for many years, but has recently been developing at a rapid pace.

The first reported use of a gel dosimeter was in 1950 by Day and Stein, using the colorimetric dye methylene blue [1]. Andrews, et al. explored chloral hydrate and trichloroethylene in agar [2]. Stein and Tomkiewicz later investigated gelatin with ferricyanide; the radiation induced oxidation of ferricyanide was well characterized and known to produce colorimetric changes [3,4]. Fricke-type gel dosimeters, named after the discoverer of the radiation induced ferric to ferrous reaction, gained significant interest following study by Gore and King using magnetic resonance (MR) imaging [5]. Investigations in 1990s introduced polymer systems which, at a basic level, consist of monomers in solution that polymerize in response to radiation [6]. Studies in the 21st century have yielded plastic leuco-dye dosimeters containing a colorless dye that reacts with free-radicals to produce color in proportion to energy deposition [7], and most recently, a polymer radio-fluorogenic gel (RFG) dosimeter, where a radio-fluorogenic monomer is activated as it is taken up into a polymer chain [8].

The above-referenced gel dosimeters all have features, often related to fabrication, limiting widespread application in radiotherapy [9]. With Fricke-type systems, the mobility of the ferrous ions allows rapid diffusion of the radiation product and subsequent loss of spatial information. Early polymer system required an evacuated glove box. Current formulations utilize an oxygen scavenger, but still suffer from monomer toxicity, although efforts have been made to minimize this [10]. Leuco-dyes are formed by mixing part-A (pre-polymer solution) and part-B (dye, catalyst, and radical initiator) solutions in a mold. Proper formation requires control of temperature and pressure. Polymer-RFG production involves a nitrogen-flushed glove box and pre-irradiation prior to use as a dosimeter. With strict fabrication methods and sometimes toxic constituents, the hunt for the ideal sensor element and gel substrate continues.

Two natural gel substrates are agarose and gelatin. Gelatin is a derivative of bovine or porcine collagen: the primary element of skin, bone, and connective tissue. Agarose is a polysaccharide isolated from agar with highest gelling potential: agar is derived from seaweed. Gelatin and agarose are both capable of creating hydrogels with low percentages of gelling agent. However, agarose is opaque and induces light scattering, while gelatin is relatively translucent. The opacity of agar makes it less than ideal for optical analysis. The clarity and transparency of gelatin are strongly dependent on raw material history, purity, and preparation. Commercial gelatin consists of tropocollagen rods in the order of 300 nm in length with 1.5 nm diameter [11]. Raw collagen is processed with acid or base solutions yielding “Type A” (hydrogen chloride) or “Type B” (sodium hydroxide). Type A is denser than type B, with a greater intrinsic viscosity [12]. Gelation speed also affects rigidity, with structure being a function of formation temperature. Slow gelation yields increased organization and orientation of chain elements with greater lateral bonding, resulting in the formation of fine well-ordered lattices [13]. Additionally, gelation is not susceptible to ionic effects [11]. Originating from mammalian tissue with well-understood mechanisms of gelation, gelatin presents as an attractive substrate for exploration of optically active sensors.

Radio-fluorogenic sensors are chemical elements that allow for dosimetry and quantification of energy deposition from of ionizing radiation through measurement of molecular fluorescence. Fluorescent detection methods are particularly promising because of their ability to form selective high-resolution images. Initially reported by Day and Stein in 1949, fluorescence spectroscopy can be used to determine absorbed dose in aqueous solutions of aromatic compounds [14,15,16]. Ionizing radiation initiates radiolysis of water yielding hydroxyl free radicals that hydroxylate aromatic compounds via electrophilic substitution. Numerous aromatic compounds are recognized as radio-fluorogenic, with hydroxylation producing fluorescent products. The first fluorescent sensor investigated for radiation dosimetry was aqueous benzoic acid [16]. Other potential sensors are terephthalic, trimesic, and pyromellitic acid [17,18,19]. Each improved the yield of fluorescent products by restraining positions on the aromatic compounds for substitution by hydroxyl radicals. However, each of the single-ring aromatic compounds possesses excitation wavelengths in the range of ultra-violet (UV) light, with a limited depth of penetration in a gel. This is the result of Rayleigh scattering of light, proportional to 1/λ^4^, resulting in rapid reduction of transmission for shorter wavelengths.

Because of their macromolecular nature, organic gels are naturally turbid; thus, it is preferable to use longer excitation wavelengths with a greater range of penetration. Fluorescence of aromatic compounds is due to their conjugated system of alternating single and double-bonds; pi-orbital overlap allows for delocalization of electrons. Larger conjugated systems require less energy for delocalization or excitation [20]. Therefore, selection of a multi-cyclic radio-fluorogenic sensor would provide the most attractive fluorescent product, ideally with excitation from visible light. Multi-cyclic coumarin-3-carboxylic acid (C3CA) is a promising sensor candidate.

**Figure 1:**
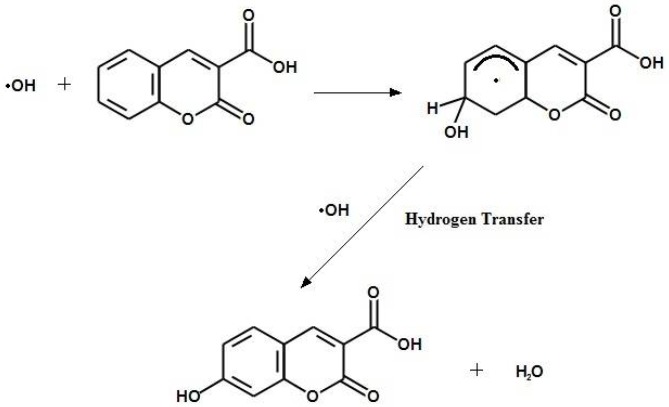
Hydroxyl radicals react with C3CA to yield umbelliferone through hydrogen abstraction, transfer, and substitution.

Aqueous C3CA has been identified as a chemical dosimeter with potential for application in radiotherapy; it demonstrates favorable traits, including linear dose response, reproducibility, and long-term stability [21]. The radio-fluorogenic mechanism of C3CA has been studied in aqueous solution [22]. Positive features of C3CA include high solubility in aqueous solutions, simple organic composition, and favorable excitation and emission spectra. C3CA reacts with hydroxyl radicals to yield the fluorescent product, 7-hydroxycoumarin-3-carboxylic acid (umbelliferone), Figure 1.

Optical imaging of biomarkers is an active area of study with several investigators exploring the use of C3CA labels for radiometric assessment [23,24,25]. The present investigation explored C3CA in gelatin as a potential RFG dosimeter. Concentration effects of C3CA were studied, and the influence of pH buffers was investigated with respect to fluorescent yield. Radiation response was examined subject to dose, rate, energy, and type for megavoltage electron and photon energies.

## 2. Materials and Methods

### 2.1. Chemical Preparation

Reagents were all purchased from Fisher Scientific (Baltimore, MD, USA): 98% C3CA (Acros Organics, Baltimore, MD, USA) and 99% umbelliferone (Infodine Chemical Company; Hillsborough, NJ, USA), sodium bicarbonate, sodium hydroxide, phosphate buffered saline, and food grade porcine type A gelatin (bloom strength 260, pH 5, and viscosity 40). All solutions were prepared with water from an EASYpure water purification system (Barnstead International; Boston, MA, USA). Large volumes of RFGs were prepared and aliquots separated, irradiated, and analyzed. Individual aliquots were stored at low temperature (5 °C) between processing steps to inhibit microbial growth.

Preparation was as follows: Gelatin was “wet” to allow for effective dispersion. Seven percent by weight gelatin was placed into a beaker to soak for 20 min, with half the total volume of water. In a separate beaker, C3CA was brought into solution by bringing a small volume of water containing C3CA to boil. The C3CA solution was then added, along with the remaining portion of water, to the “wet” gelatin. The temperature of the RFG solution was then raised to 35 °C. It is important for the temperature to remain below 40 °C to prevent denaturation of the gelatin. The RFG was maintained at 35 °C for approximately 90 min, or until it presented as optically clear and free of visible colloidal structures. The RFG was then removed from heat and pipetted into poly-methyl-methacrylate (PMMA) cuvettes. Cuvettes were left to cool overnight at ambient temperature.

For studies with different pHs, umbelliferone was used to mimic the radio-fluorogenic product. Seven percent gelatin by weight and 0.9 mM C3CA and 0.1 mM umbelliferone solutions were prepared with deionized water (pH 6.0), 0.05 mM sodium bicarbonate and 0.1 mM sodium hydroxide (basic reagents, pH 10), and stock phosphate buffered saline (PBS, pH 7.4). For dose response investigations, RFGs with various coumarin concentrations (1 mM, 5 mM, 10 mM, and 20 mM) were prepared using basic reagents.

### 2.2. Irradiation and Analysis

**Figure 2:**
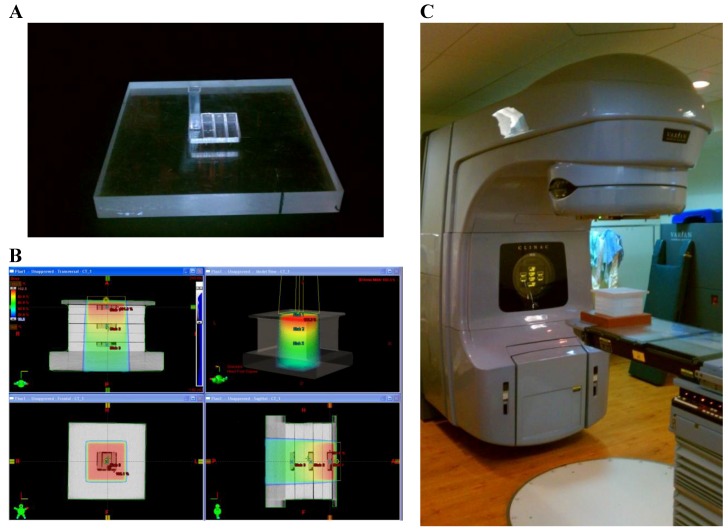
(**A**) Polystyrene phantom plate with void for irradiation of cuvettes; (**B**) dose calculation of phantom (heat map represents relative dose); (**C**) medical linear accelerator used for irradiations.

Irradiations were conducted with a high-energy medical linear accelerator (Varian Medical Systems; Palo Alto, CA, USA). Samples were irradiated with two nominal megavoltage (MV) photon energies (6 & 23 MV) and one electron energy (9 MeV). Irradiations were conducted with samples placed in a polystyrene phantom containing a void for 4 cuvettes. The phantom was designed to provide favorable geometry for the establishment of electronic equilibrium, providing an even distribution of imparted energy. A computed tomography (CT) scan was performed on the phantom, and images were imported into the Eclipse treatment planning system (Varian Medical Systems; Palo Alto, CA, USA) for calculation of dose, Figure 2.

Instrumental analysis was conducted with a Cary Eclipse fluorescence spectrophotometer (Varian, Inc.; Pal Alto, CA, USA). Excitation and emission slit widths were set to 5 nm, emission scans were performed, and peak emission values recorded and plotted. Dose response was measured by observing the intensity of 445 nm emissions.

## 3. Results

### 3.1. pH Response

**Figure 3:**
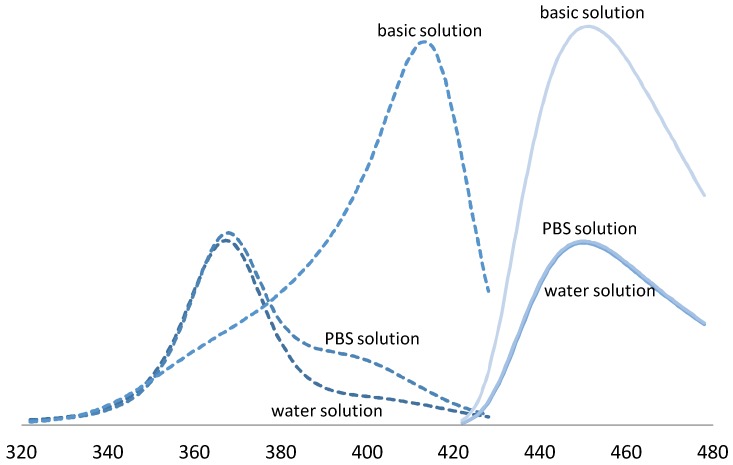
Fluorescence properties of coumarin-gelatin RFG dosimeter, excitation (dashed-lines) and emission (solid-lines) spectra for solutions of 7% gelatin with 0.9 mM C3CA and 0.1 mM umbelliferone. Curves are labeled in the graph. *X*-axis represents wavelength of emitted and collected light, *Y*-axis is not shown but represents an arbitrary unit of light intensity.

The influence of pH buffers on fluorescent response was examined. Results showed a doubling of fluorescent yield in basic solution (pH 10). A spectral shift of the excitation maxima was also observed. Specifically, peak excitation shifted from 365 nm in solutions prepared with water compared to 405 nm in solutions prepared with basic reagents, Figure 3.

### 3.2. C3CA Concentration

**Figure 4:**
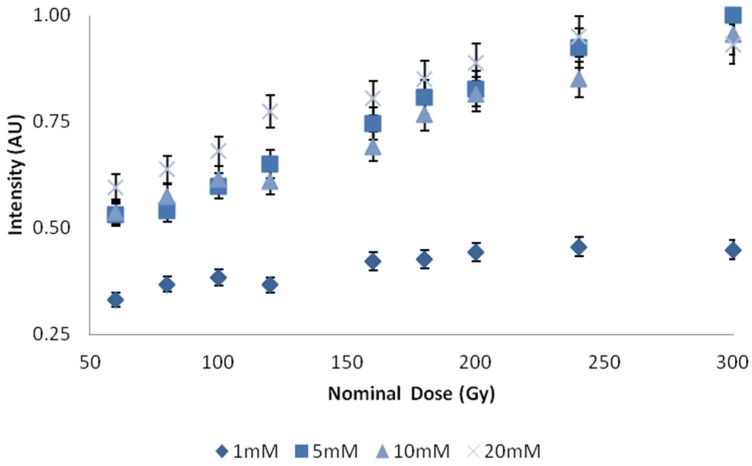
Dose plotted against intensity for concentrations of C3CA in 7% gelatin. *X*-axis represents central axis dose for the depth of cuvette, *Y*-axis, represents an arbitrary unit of light intensity. Error bars represent a 5% variation from the mean; calculated standard deviation from *n* = 4 samples per data point, less than 1%.

Varying the concentration of C3CA in solutions prepared with basic reagents (pH 10) demonstrated a significantly increased response with concentrations 5 mM and greater, Figure 4. Repeated measures, using four samples for each data point, demonstrated standard deviations of less than 1%.

### 3.3. Dose Response

**Figure 5:**
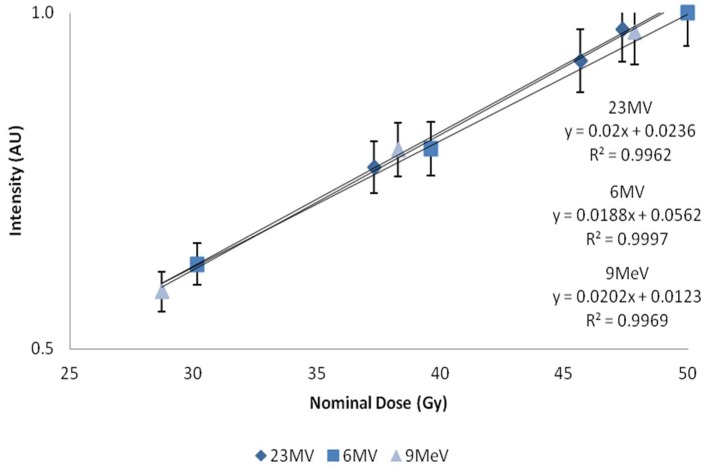
Nominal dose plotted against intensity. *X*-axis represents central axis dose for the depth of cuvette, and *Y*-axis the normalized fluorescent intensity. Error bars represent a 5% variation from the mean; calculated standard deviations from *n* = 4 samples per data point, less than 1%.

**Figure 6:**
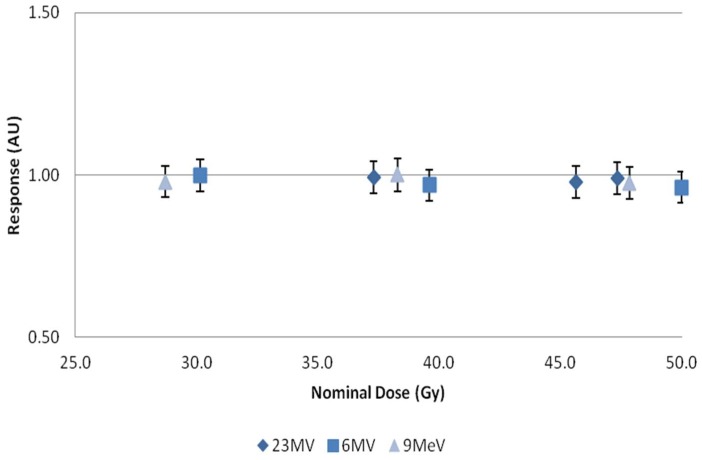
Normalized response plotted against nominal dose for 23 MV, 6 MV, and 9 MeV beams. *X*-axis represents central axis dose for the depth of cuvette, and *Y*-axis the normalized dose response. Error bars represent a 5% variation from the mean; calculated standard deviations from *n* = 4 samples per data point, less than 1%.

**Figure 7:**
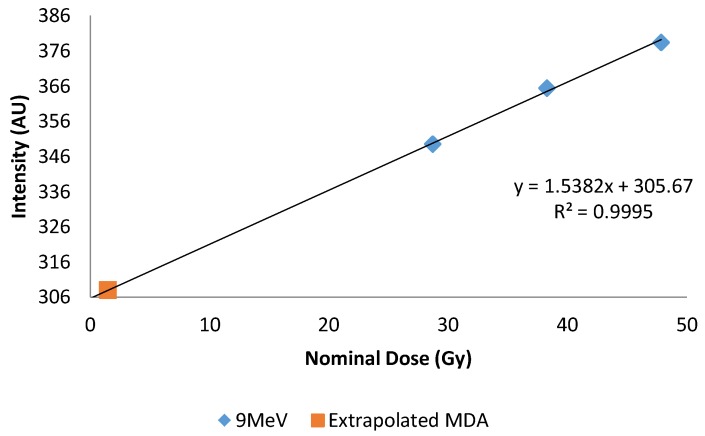
Plot of 9 MeV dose response plotted with extrapolated MDA. *X*-axis represents central axis dose for the depth of cuvettes. *Y*-axis is the normalized fluorescent intensity.

Dose response was determined by plotting 445 nm emissions versus dose, Figure 5. A linear response was observed in the range investigated, independent of type (photon or electron) and energy, Figure 6. Using a definition of three times the standard deviation of the background, the minimum detectable amount (MDA) was extrapolated from 9 MeV electron data, and found to be 1.5 Gy, Figure 7.

## 4. Discussion

Basic solutions (pH 10) of the gelatin-coumarin dosimeter were observed to double emission intensity and shift the peak excitation wavelength from 365 nm to 405 nm. Transition between excited and ground states, i.e., the energy gap, is known to be influenced by the micro-environment through molecular motion, collision, rotational and translational diffusion, and the formation of complexes. Smaller quantum yields are observed with large energy gaps due to the availability of alternative relaxation pathways. The observed increase in quantum yield is consistent with previous studies in aqueous solution; however, the wavelength shift was greater than previously observed (385 nm) [26]. The increased spectral shift may be an unexplored result of interactions with gelatin.

Dose response was notably more pronounced for concentrations of C3CA above 5 mM. A concentration of 10 mM was selected for further study, but 5 mM may have been a better selection; 10 mM values appear to demonstrate decreased intensities, most likely due to the inner filter effect. Observations show an independent linear response with respect to dose, energy, and type of ionizing radiation (electron and photon). With respect to type, an independent response is expected, since photon dose is predominately deposited by delta rays, secondary electrons. A dose rate dependency was observed, which was consistent with other findings [21]. This dose rate dependency has previously been suggested to be the result of metallic impurities in C3CA. Future work should explore successive distillations to remove impurities and an expanded dose range.

The potential of RFGs for determination of spatial dose distributions has been previously demonstrated [27,28], and the use of planar laser-induced fluorescence (PLIF) has been suggested and demonstrated to yield high-resolution images [29,30]. Independent investigators have recognized PLIF as a valid method, and are currently developing a reader system [31]. However, it is our belief that the finest imaging resolution for RFG gel dosimetry will be achieved by applying methods of two-photon excitation microscopy, allowing the use of longer wavelength light possessing a greater depth of penetration. With recent reports of an RFG using a nanoclay substrate and advances in the fabrication of 3D printable phantom materials, further study is encouraged [32,33].

## 5. Conclusions

Our report shares some studies of a novel coumarin-gelatin RFG dosimeter. We found that a significant increase in quantum yield can be achieved with a coumarin-gelatin RFG in basic solution. Gels had a linear dose response with potential for application in the therapeutic dose range. RFG dosimeters warrant further study due to the selective high-resolution images that can be obtained with fluorescent analysis.
